# Impact of group size and habitat disturbance on parasitic infection in free-ranging proboscis monkeys

**DOI:** 10.1016/j.ijppaw.2026.101221

**Published:** 2026-03-19

**Authors:** Muhammad Nur Fitri-Suhaimi, Liesbeth Frias, Elke Zimmermann, Primus Lambut, Joseph Tangah, Henry Bernard, Vijay Subbiah Kumar, Ikki Matsuda

**Affiliations:** a Wildlife Research Center of Kyoto University, 2-24 Tanaka-Sekiden-cho, Sakyo, Kyoto, 606-8203, Japan; b Department of Infectious Diseases and Public Health, Jockey Club College of Veterinary Medicine and Life Sciences, City University of Hong Kong, Hong Kong SAR; c Institute of Zoology, University of Veterinary Medicine Hannover, Hannover, Lower Saxony, Germany; d Sabah Wildlife Department, Wisma Muis, Kota Kinabalu, Sabah, 88100, Malaysia; e Sabah Forestry Department, Forest Research Centre, Sandakan, Sabah, Malaysia; f Institute for Tropical Biology and Conservation, Universiti Malaysia Sabah, Jalan UMS, Kota Kinabalu, Sabah, 88400, Malaysia; g Biotechnology Research Institute, Universiti Malaysia Sabah, Jalan UMS, Kota Kinabalu, Sabah, 88400, Malaysia; h Chubu Institute for Advanced Studies, Chubu University, Kasugai, Aichi, Japan

**Keywords:** Host-parasite interactions, Anthropogenic impact, Primates, Social organization, Zoonotic risk, Wildlife disease ecology

## Abstract

Group-living primates experience the benefits and costs associated with sociality, including an elevated risk of parasite transmission. However, the relative influence of group type (i.e., social structure), group size, and habitat disturbance on parasitic infection remains unclear, particularly in Southeast Asian primates. In this study, the abundance of intestinal parasites in proboscis monkeys (*Nasalis larvatus*) inhabiting a riverine forest along the Menanggul River, Sabah, Malaysian Borneo, was investigated. Fecal samples (n = 160) were collected from one-male-multifemale and all-male groups in areas with varying levels of anthropogenic disturbance, with efforts made to ensure that each sample originated from a different individual. In addition, the effects of group type, group size, and sampling location on parasite abundance were evaluated using fecal egg counts and Bayesian models. Three dominant parasite species groups (*Trichuris* sp., *Strongyloides fuelleborni*, and *Oesophagostomum aculeatum*) with an overall infection prevalence of 81.25% were identified. Results showed that group type did not significantly affect parasite abundance. However, group size showed a positive correlation with the abundance of *Trichuris* sp. and a negative correlation with *S. fuelleborni* and *O. aculeatum*. In addition, our models revealed that the infection load of *Trichuris* sp. decreased with increasing distance from the river mouth, which was used as a proxy for a disturbance gradient, whereas *O. aculeatum* exhibited higher infection load at greater distances, indicating lower prevalence in more disturbed downstream areas. Thus, parasite abundance in proboscis monkeys may be shaped by social and environmental factors, with taxa-specific responses likely reflecting differences in environmental persistence and transmission ecology.

## Highlights

• Examines intestinal parasite patterns in free-ranging proboscis monkeys across a riverine forest.
• Parasite taxa showed contrasting associations with host group size.
• Environmental and anthropogenic gradients influenced parasite abundance in a taxon-specific manner.
• Results highlight complex interactions between social and environmental drivers of wildlife parasitism.

## Graphical abstract

**Figure:**
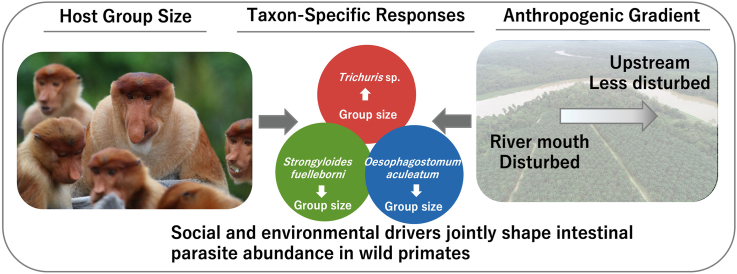

## 1 Introduction

Highly social animals such as primates exhibit complex group dynamics that influence their health, behavior, and interactions with pathogens (Kappeler et al., 2015). Although group-living promotes social support and cooperative behaviors that can mitigate some health risks (Silk, 2007), it also imposes costs, including an increased potential for pathogen transmission and heightened susceptibility to disease caused by social stress (Altizer et al., 2003). Thus, the relationship between sociality and disease risk is complicated. Factors such as group structure, contact patterns, and social hierarchies all play key roles in determining infection dynamics (Nunn et al., 2015). Notably, this relationship is bidirectional in primates. While social factors such as group size and composition influence parasite transmission, parasite infection can also feed back to shape host social behavior. Consequently, infection prevalence and intensity can affect social interaction and group dynamics (Vitone et al., 2004; Nunn, 2012; Rushmore et al., 2017).

Apart from social dynamics, anthropogenic environmental changes, such as deforestation and provisioning, can also influence parasite transmission by altering host movement patterns, population densities, and contact rates (Gillespie and Chapman, 2006; Becker et al., 2018). These ecological modifications can create conditions that either facilitate or hinder parasite spread, depending on the nature and extent of human-induced disturbances. Therefore, understanding the mechanisms by which environmental, social, and ecological factors contribute to parasitic infections in social animals is crucial for developing effective conservation and management strategies (Chapman et al., 2005; Gillespie and Chapman, 2006).

On the contrary, research on parasitic infection and sociality in wild primates has yielded inconsistent findings, while the effects of anthropogenic environmental changes on parasitic infections in primates have been relatively consistent (Gillespie and Chapman, 2006). The relationship between parasite load and group size is complex, as it depends on various factors, such as host susceptibility and environmental conditions (e.g. Chapman et al., 2009; Klaus et al., 2018). Although larger groups might increase the likelihood of infection because of higher social interactions, group mobility and seasonal changes may reduce the risk of parasite transmission (e.g., increased precipitation during the rainy season washing away feces and parasites), thereby complicating the host–parasite dynamics (Nguyen et al., 2015; Radespiel et al., 2015; Knauf and Jones-Engel, 2020).

Although knowledge of parasitism in wild primates is expanding, it remains limited, particularly in Southeast Asia (Hopkins and Nunn, 2007; Cooper and Nunn, 2013). In Borneo, which is a highly biodiverse island in Southeast Asia, numerous endemic species are experiencing population declines (Wilcove et al., 2013), and the large arboreal proboscis monkey (*Nasalis larvatus*) inhabiting mangroves, peat swamps, and riverine forests is no exception to this trend (Lhota et al., 2019; Bernard et al., 2025). Several studies have provided insights into primate–parasite interactions in Sabah, including the proboscis monkey. This primate has unique parasite associations (Hasegawa et al., 2020) and infection dynamics that reflect its specialized ecological adaptations (Klaus et al., 2017) and interactions with sympatric primates (Frias et al., 2019, 2021). Previous studies have reported a high prevalence of helminth eggs in fecal samples, identifying five major parasite groups, with co-infections being common (Salgado-Lynn, 2010; Klaus et al., 2017; Frias et al., 2021). In addition, parasite species richness has been reported to be significantly higher in semi-wild populations than in wild ones. This increase likely results from the high levels of human disturbance, which can alter the mechanism by which parasites are transmitted. By contrast, group type and group size have been reported not to significantly affect parasite richness (Klaus et al., 2018). Despite the increasing knowledge of parasitic infections in proboscis monkeys, the fine-scale variation in infection patterns –across individuals or groups inhabiting distinct microhabitats and experiencing different levels of anthropogenic disturbance– remains unclear.

In this study, the abundance of intestinal parasites in proboscis monkeys was investigated, with particular emphasis on the effects of group type, group size, and habitat disturbance. Fecal samples collected over a 1-year period were analyzed to identify the parasites present and to examine the mechanism by which group size and social structure (i.e., group type: one-male-multifemale or all-male groups) influence parasite loads in a forest subjected to varying degrees of anthropogenic disturbance. Considering that proboscis monkeys exhibit seasonal variation in foraging behavior, ranging patterns, and intergroup spacing at our study site (Matsuda et al., 2009a, 2009b, 2010a), a year-round sampling approach may provide a comprehensive assessment of parasite dynamics. Thus, extended sampling could reveal a positive correlation between group size and parasitic infection, which is contrary to previous findings (Klaus et al., 2018). In addition, the study forest along the tributary river exhibits varying degrees of anthropogenic disturbance between the upstream and downstream areas. In particular, downstream areas are more strongly influenced by tourism-related boat traffic, human activity, and proximity to oil palm plantations, whereas upstream areas retain relatively higher plant diversity (Jose et al., 2024). The impact of habitat disturbance on parasite abundance can be assessed by comparing the fecal samples collected from individuals across these areas. Given that research in our study area has documented alterations in the gut microbiota of proboscis monkeys living in disturbed versus undisturbed areas (Jose et al., 2024), we hypothesize that parasite abundance would also differ between these environments because of human-induced disturbances.

## 2 Methods

### 2.1 Study site and subjects

This study was conducted in a riverine forest along the Menanggul River, a tributary of the Kinabatangan River, in Sabah, Malaysian Borneo (118°30′ E, 5°30′ N). The study site has a mean daily temperature range of 24°C–30 °C and an annual average precipitation of 2474mm (Matsuda et al., 2019). The river exhibits daily water-level fluctuations of approximately 1 m, while seasonal inundation results in an average increase exceeding 3 m (Matsuda et al., 2010b). The study area spans 4 km along the Menanggul River and supports a minimum population of 200 proboscis monkeys, forming a multilevel society composed of one-male-multifemale groups and all-male groups, which frequently assemble in the riverside (Matsuda et al., 2024). The riparian zone primarily consists of secondary forest in the southern portion, while much of the northern portion has been converted to oil palm plantations, except for a narrow, protected riparian strip. Accordingly, in this tributary, forests extending upstream of the river mouth exhibit a higher plant diversity (Jose et al., 2024). The proboscis monkeys in the study area are well habituated to human presence, as the Menanggul River has been a major tourist attraction for over a decade and serves as a frequent route for boats and observers.

### 2.2 Sample collection

Between June 2015 and April 2016, boat surveys were conducted in the late afternoon on approximately 2–3 days per week to locate proboscis monkeys, as they typically return to riverside trees to sleep (Matsuda et al., 2011), and to record group composition and the GPS coordinates of sleeping locations. The following morning, before the monkeys awakened, these sleeping locations were revisited. Given that proboscis monkeys often defecate just before moving into the forest, the area around their sleeping sites was carefully searched for fresh feces after the monkeys had departed their sleeping trees. Multiple one-male-multifemale groups often remained close to one another along the river, making it challenging to determine to which group the feces found on the ground belonged. Therefore, we inferred the group associated with the feces based on the location of the feces and the group observed in the previous day's survey. Only fecal samples presumed to originate from adult individuals were included, based on fecal size, which allows for differentiation between adults and immature individuals despite the challenges in distinguishing feces from neighboring groups (Matsuda et al., 2014). Consequently, between June 2015 and April 2016, 160 fecal samples were collected. Individual identity was subsequently confirmed through genetic analyses, which verified that the samples originated from genetically distinct individuals (Matsuda et al., 2024). At least nine groups were identified in the study area, although the number of groups these fecal samples were collected from remains unclear, as group identification was not complete at the time the samples were collected (Matsuda et al., 2024). The fecal samples were preserved in tubes with 70% ethanol.

### 2.3 Parasite detection and identification

A modified protocol was used to concentrate parasite eggs via formalin-ethyl acetate sedimentation, and fecal samples were analyzed using the sequential sedimentation–flotation method (Frias et al., 2021). The final pellet was resuspended in saline, and 1 mL of aliquot was transferred into a vial and placed on a magnetic stirrer to ensure thorough homogenization during analysis. In estimating the number of eggs per gram of feces (EPG) in each sample, an aliquot was collected from the homogeneous suspension and placed in a McMaster counting chamber for examination at 100 × magnification. EPG was calculated on the basis of the average of five replicate counts of parasite eggs observed within the chamber's grid, using the known weight of fecal sediment in a 0.15 mL volume. After quantifying the eggs, each sample was centrifuged again; the supernatant was discarded, and the concentrated pellet was resuspended in Sheather's solution with a specific gravity of 1.27. Two slides were examined to reduce the likelihood of overlooking less abundant helminth eggs. Parasite identification was performed using standard identification keys (Modrý et al., 2018).

### 2.4 Parasite prevalence and abundance

The prevalence of a given parasite was calculated as the percentage of hosts infected by that parasite. Parasite abundance was estimated for each sample using the EPG rounded to the nearest integer as a proxy measure, following the terminology of Bush et al. (1997).

### 2.5 Factors influencing parasite abundance

A Bayesian model was developed using the “brms” package (Bürkner, 2018) to evaluate the mechanism by which group type (one-male-multifemale or all-male groups), group size, and fecal sampling locations affect parasite abundance. Sampling locations were represented by distance from the river mouth to upstream locations, which served as a proxy for the disturbance gradient along the river system. In our analysis, parasite abundance was used as a dependent variable and modelled using a Poisson distribution with a log link. Group type was incorporated as a group-level effect, whereas group size and the distance of the sampling location to the river mouth were included as continuous variables. All models were controlled for host group identity. Distinct groups were not consistently identifiable throughout the study period. Therefore, we assigned samples to groups based on their spatial and temporal proximity along the river. This approach was guided by observations: the mean distance between consecutive sleeping sites for each group was approximately 350 m in the study area (Matsuda et al., 2024). In addition, changes in food availability over time can influence spatial patterns along the river (Matsuda et al., 2010a). Based on these criteria, two samples collected within 350 m of each other and during the same month were classified as originating from the same group. Groups with fewer than three observations were excluded from the analysis.

Weakly informative priors were assigned to population-level effects, following a Student's t-distribution. The posterior distributions of the model parameters were estimated using Markov chain Monte Carlo methods. For this purpose, four chains were constructed, each consisting of 2000 steps, with 1000-step warm-up periods. Consequently, a total of 4000 steps were retained to estimate the posterior distributions. The independent continuous variables were scaled to aid model fitting and coefficient interpretation. Chain convergence was assessed visually, and the potential scale reduction factor (Rhat) values were examined to ensure they were less than or equal to 1 for all parameters (Bürkner, 2018). This modeling process was repeated for the three parasites identified within the host population.

## 3 Results

### 3.1 Parasite prevalence and abundance

Parasites were detected in 130 of the 160 analyzed samples, yielding an overall parasite prevalence of 81.25%. The eggs of *Trichuris* sp. (78.12%, n = 125), *Strongyloides fuelleborni* (10.62%, n = 17), *Oesophagostomum aculeatum* (5%, n = 8), *Enterobius* (*Colobenterobius*) *serratus* (1.25%, n = 2), and *Spirurida* sp. (0.63%, n = 1) were identified. The mean abundance of the top three prevalent parasites was recorded at 168.2 EPG (range: 0–984), 9.41 EPG (range: 0–650), and 2.83 EPG (range: 0–109), respectively.

### 3.2 Factors influencing parasite abundance

**Fig. 1:**
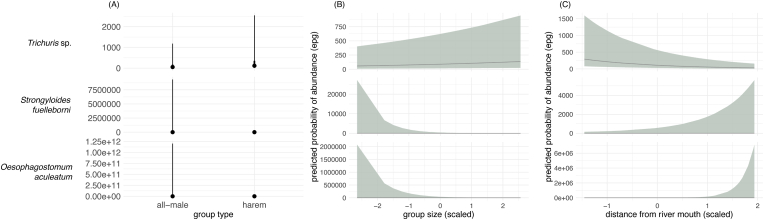
Posterior distributions of the differences in parasite abundance for *Trichuris* sp. (top), *S. fuelleborni* (middle), and *O. aculeatum* (bottom) in relation to host group type (A). The green shaded areas represent the posterior distributions, with the black dot indicating the median estimate and the black bold horizontal line representing the 99% credible interval. The vertical dashed line at zero represents no difference in infection rates between groups. Predicted probability of abundance for *the same parasite groups* in relation to host group size (B) and sampling location represented by the distance from the river mouth (used as a proxy for disturbance gradient) (C). Credible intervals are set at 89%.

Of the 160 fecal samples examined, 124 were included in the Bayesian analyses after excluding samples from groups with fewer than three observations, as these could not be reliably incorporated into the group-level models. The type of host group did not have a statistically significant effect on parasite abundance across any of the parasite groups (Fig. 1A). However, host group size emerged as an important predictor of parasite abundance, with varying effects across parasite groups. The abundance of *Trichuris* sp. was positively correlated with group size, whereas that of *S. fuelleborni* and *O. aculeatum* was negatively correlated with group size (Fig. 1B). In addition, our models indicated that the distance from the river mouth to upstream locations, used as a proxy for a disturbance gradient, markedly influenced the parasite abundance for *Trichuris* sp. and *O. aculeatum*. In particular, as the distance from the river increased, the abundance of *Trichuris* sp. decreased, whereas *O. aculeatum* exhibited an opposite trend, with higher counts linked to greater distances from the river mouth (Fig. 1C). A summary of the results from the models is provided in Table 1.

**Table 1:** Summary of fitted models.

	Parameter	Est.	Est. error	95% CI (Lower)	95% CI (Upper)	Rhat	Bulk ESS	Tail ESS
*Trichuris* sp.	*Random Effects*							
	sd (Intercept)	0.92	0.22	0.6	1.44	1	1600	2336
	*Fixed Effects*							
	Intercept	4.32	0.51	3.29	5.32	1	3515	4008
	group type	0.81	0.55	−0.27	1.93	1	3151	3511
	group size (scaled)	0.16	0.01	0.14	0.19	1	6937	4494
	distance to river (scaled)	−0.7	0.14	−0.97	−0.44	1	2949	3362
*S. fuelleborni*	*Random Effects*							
	sd (Intercept)	5.74	1.67	3.37	9.71	1	2691	3424
	*Fixed Effects*							
	Intercept	−1.68	3.25	−8.21	4.77	1	3101	3670
	group type	−1.09	3.7	−8.69	5.88	1	2916	3602
	group size (scaled)	−1.62	0.12	−1.86	−1.38	1	9301	5682
	distance to river (scaled)	1.03	0.71	−0.35	2.44	1	5057	4881
*O. aculeatum*	*Random Effects*							
	sd (Intercept)	10.54	3.96	5	20.28	1	2116	3425
	*Fixed Effects*							
	Intercept	−5.12	6.63	−18.87	7.67	1	3150	3920
	group type	−0.47	7.58	−16.08	14.32	1	2693	3620
	group size (scaled)	−1.48	0.19	−1.87	−1.11	1	7942	5445
	distance to river (scaled)	3.46	1.4	0.71	6.27	1	3953	4708

## 4 Discussion

In this study, the relationship among social structure (i.e., group type: one-male-multifemale and all-male groups), the degree of anthropogenic habitat disturbances within a 4-km stretch along the river, and parasite load in free-ranging proboscis monkeys was examined. A high prevalence of intestinal parasites was observed (81.25%), which is consistent with the previous prevalences recorded for proboscis monkeys along the Kinabatangan River in Sabah [i.e., 96.6% in Salgado-Lynn (2010), 92.3% in Klaus et al. (2017), and 80.2% in Frias et al. (2021)] and in Bako National Park in Sarawak [i.e., 95.4% in Adrus et al. (2019) ]. The predominant parasite groups in this study included *Trichuris* sp., *Strongyloides fuelleborni*, and *Oesophagostomum aculeatum*, which have also been identified as dominant in other fecal analyses of proboscis monkeys, in wild and captive settings (Table 2), reinforcing a consistent pattern of parasitic infections across different populations. Nonetheless, given the substantial differences in the diets of proboscis monkeys between riverine and mangrove forests (Matsuda et al., 2019), habitat-linked variation in food use may also influence parasite exposure. Because habitat type determines not only the food resources available to proboscis monkeys but also forest height and frequency of ground use or sediment contact during foraging (Feilen and Marshall, 2014, 2020; Atmoko et al., 2021), these ecological differences may affect transmission dynamics of environmentally transmitted parasites. Thus, whether similar trends in parasite prevalence are observed among proboscis monkeys across different habitat types on the island of Borneo should be examined in the future.

**Table 2:** Comparison of the parasite species detected and parasite prevalence (%) in fecal samples of proboscis monkeys in each study.

	Bekantan Rescue Center Sahabat Bekantan Foundation (Rahman et al., 2023)	Surabaya Zoo (Ramadhani et al., 2024)	Labuk Bay Proboscis Monkey Sanctuary (Klaus et al., 2017, 2018)	Bako National Park (Adrus et al., 2019)	LKWS^a^ (Lots 1-7)(Frias et al., 2021)	LKWS (Lot 6)(Klaus et al., 2017, 2018)	LKWS (Lots 1-4)(Salgado-Lynn, 2010)	LKWS (Lot 4)(this study)
**Forest type**	N/A	N/A	Mangrove	Mangrove	Riverine forest	Riverine forest	Riverine forest	Riverine forest
**Living-condition**	Captive	Captive	Semi-free ranging	Free-ranging	Free-ranging	Free-ranging	Free-ranging	Free-ranging
**No. of individuals**	3	<27	<195	Unknown	Unknown	< ca. 200	Unknown	160
**No. of samples**	60	20	90	65	91	652	146	160
**Cestodes**								
*Diphyllobotrium* sp.	–	–	–	1.5	–	–	–	–
*Dipylidium*-like morphs	–	–	–	–	–	–	9.6	–
*Hymenolepis nana*	–	5.0	–	–	–	–	–	–
*Hymenolepis* sp.	–	–	–	3.1	–	–	–	–
*Taenia* sp.	–	–	–	–	–	–	28.8	–
**Nematodes**								
*Anatrichosoma* spp.	–	–	2.8	–	–	1.4	2.1	–
*Ascaris lumbricoides*	–	–	2.2	–	–	8.6	–	–
*Ascaris* sp.	–	5.0	–	30.8	–	–	67.1	–
*Enterobius (Colobenterobius) serratus*	–	–	–	–	1.25	–	–	1.25
*Enterobius* spp.	–	–	–	–	–	5.5	–	–
*Oesophagostomum aculeatum*	–	–	–	–	–	–	–	5.0
*Oesophagostomum* sp.	5.0	–	31.4	33.8	–	22.8	–	–
oxyurid	–	–	–	–	–	–	4.1	–
*Physaloptera* sp.	–	–	–	3.1	–	–	–	–
*Spirurida* sp.	–	–	–	–	2.1	–	–	0.63
strongylid	–	–	4.6	–	–	–	45.2	–
Strongylida	–	–	43.3	–	29.6	3.1	–	–
*Strongyloides fuelleborni*	–	–	–	–	–	–	–	10.6
*Strongyloides stercoralis*	–	–	–	4.6	–	–	–	–
*Strongyloides* spp.	–	5.0	65.6	21.5	20.8	32.7	5.5	–
*Trichostrongylus* spp.	10.0	–	36.0	9.2	–	48.5	–	–
*Trichuris trichiura*	15.0	–	–	44.6	–	–	–	–
*Trichuris* spp.	–	85.0	21.8–47.1	35.4	80.2	82.1	91.8	78.1
**Trematodes**								
*Fasciola* sp.	–	–	–	1.5	–	–	–	–
*Schistosoma* sp.	–	–	–	1.5	–	–	–	–

a Lower Kinabatangan Wildlife Sanctuary.

However, no significant association was found between parasite abundance and group type (i.e., one-male-multifemale and all-male groups) in proboscis monkeys in this study, consistent with previous findings (Klaus et al., 2018), suggesting that sex differences may have a limited influence on parasitic infection. This interpretation is further supported by a study conducted at the same site that examined gut microbial composition in fecal samples and found no significant sex differences (Jose et al., 2024). Given that gut microbiota, such as intestinal parasites, would be transmitted through direct or indirect social contact and shared use of space and food resources, the absence of sex-specific patterns in both types of organisms indicates that behavioral and spatial overlap between males and females minimizes sex-related divergence in microbial and parasite exposure.

Contrary to the findings of Klaus et al. (2018), our study revealed that group size might be a potential factor influencing parasite abundance for a particular parasite species, namely, *Trichuris* sp. This finding supports the hypothesis that larger groups may exhibit higher infection rates, probably because of increased contact rates and overlapping space use among individuals (Snaith et al., 2008; Rifkin et al., 2012; Kappeler et al., 2015). Although the exact mechanism remains unclear, a comparable trend was observed in the gut microbial diversity in the same population. A larger group size was associated with greater alpha diversity (Jose et al., 2024), suggesting that higher host density may increase exposure to intestinal parasites and microbes. This association may reflect the increased direct interactions among individuals and result from enhanced environmental contamination in frequently reused areas. Given that all the parasites identified in this study could be transmitted via fecal-oral routes, the accumulation of feces in shared sleeping or foraging sites could be a key factor driving infection. Limited territoriality and habitual reuse of sleeping trees by proboscis monkeys –often shared across one-male-multifemale and all-male groups in multilevel societies– likely facilitates such transmission (Matsuda et al., 2024). Furthermore, the differences in habitat structure and spatial dynamics among the study sites may explain the contrasting results reported by Klaus et al. (2018). They focused on groups inhabiting the main channel of the Kinabatangan River, where the river's width (>200 m) limits cross-group movement and shared space use. By contrast, our study site along narrow tributaries (15–20 m wide) may allow more frequent crossings and higher spatial overlap among groups (Matsuda et al., 2008, 2010a), thereby promoting indirect transmission through contaminated environments.

On the contrary, *S. fuelleborni* and *O. aculeatum* were negatively correlated with group size because of their differing ecological tolerances. *S. fuelleborni*, which is a parasitic nematode, may be more influenced by habitat disturbance or seasonal changes, with larger groups potentially reducing parasite exposure through increased host mobility and spatial separation from contaminated areas (Gillespie and Chapman, 2006; Radespiel et al., 2015). Similarly, *O. aculeatum* may require more stable environmental conditions for successful transmission, as the development and survival of free-living stages in *Oesophagostomum* spp. are influenced by factors such as temperature and related habitat conditions (Sengupta et al., 2016; Senanayake et al., 2025). The relatively undisturbed and pristine upstream habitats may therefore provide better conditions for transmission compared with downstream areas, whereas larger groups in disturbed downstream areas could hinder transmission (Kappeler et al., 2015).

More broadly, these contrasting patterns may reflect a trade-off between contamination density and spatial avoidance. Larger groups may increase fecal contamination at frequently reused sites, favoring parasites with environmentally resistant stages such as *Trichuris*. Conversely, increased host mobility and shorter residence time at specific patches may reduce exposure to parasites with more fragile free-living stages, such as *S. fuelleborni* and *O. aculeatum*. These findings indicate that host social dynamics and environmental factors must be considered, as species-specific responses shape infection patterns (Rifkin et al., 2012; Nguyen et al., 2015).

Our findings revealed a more complex relationship between parasite abundance and habitat disturbance than initially anticipated. Contrary to expectations that anthropogenic habitat disturbance would universally elevate parasite loads (Gillespie and Chapman, 2006), we observed that the impact of disturbance varied across different parasite taxa. In particular, the abundance of *Trichuris* sp. decreased with increasing distance from the river mouth, indicating that higher parasite loads occur in downstream areas with more intense human activity and tourism. Conversely, *O. aculeatum* displayed the opposite trend, with greater abundance in less disturbed upstream forests. These contrasting patterns indicate that different parasite species may respond to habitat disturbance through distinct ecological mechanisms. However, we acknowledge that distance from the river mouth serves as a proxy that likely integrates multiple environmental gradients beyond anthropogenic disturbance alone. These may include natural variation in soil composition, flooding regimes, vegetation structure, and microclimatic stability, all of which could influence parasite spatial responses observed between *Trichuris* sp. and *O. aculeatum.*

This interpretation would be consistent with differences in environmental persistence between parasite taxa, as *Trichuris* eggs are relatively resistant, whereas *O. aculeatum* may depend more strongly on stable microenvironmental conditions for successful development and transmission. Considering the elevated *Trichuris* sp. abundance in downstream areas, more disturbed habitats may not be attributable to the increased host density or spatial overlap, as proboscis monkeys in these areas do not necessarily aggregate at higher densities compared with the upstream areas. Aggregation likely occurs in upstream areas, where the river is narrower, providing a safer environment for proboscis monkeys because they can escape terrestrial predators, such as clouded leopards, by jumping to the opposite bank (Matsuda et al., 2010a). Given its greater safety from predators, this environment likely facilitates aggregation in upstream areas, making them a more favorable location for proboscis monkey groups to congregate. This interpretation is consistent with the species’ highly resilient egg morphology, which supports environmental persistence even under degraded conditions (Gillespie and Chapman, 2006; Snaith et al., 2008). Morphological robustness may enable *Trichuris* sp. to maintain transmission success across a range of habitat types, including more disturbed downstream areas. By contrast, successful transmission of *O. aculeatum* requires embryonation, hatching, and survival of infective larval stages in the environment, processes that are influenced by environmental conditions such as temperature and moisture (Rose and Small, 1980; Radespiel et al., 2015; Sengupta et al., 2016; Senanayake et al., 2025). Accordingly, the relatively pristine conditions in upstream sites likely provide a favorable microenvironment for their development.

Interestingly, these spatial patterns in parasite abundance are contrary to prior findings from the same population, which identified clear site-based differences in gut microbial composition, with lower microbial diversity in the downstream areas (Jose et al., 2024). Gut microbiota and intestinal parasites share common transmission pathways, including social contact and environmental exposure. However, their ecological sensitivities appear to diverge. In general, gut microbiota are more responsive to dietary shifts and subtle ecological changes (e.g., Amato et al., 2013; Hayakawa et al., 2018; Lee et al., 2019; Barelli et al., 2020). By contrast, parasite abundance in our study is influenced by a combination of host social dynamics (e.g., group size and space use) and species-specific environmental tolerances. This distinction emphasizes the importance of adopting a taxon-specific approach when interpreting the host–environment–parasite relationship. Furthermore, it indicates that parasites and microbiota may differentially influence the ecological and health dynamics of primates (Rifkin et al., 2012; Nguyen et al., 2015).

These contrasting patterns between gut microbiota diversity and *Trichuris* abundance may reflect differences in the ecological processes governing their assembly. Gut microbiota are strongly shaped by dietary and environmental inputs, which can be altered by habitat disturbance (Amato et al., 2013; Hayakawa et al., 2018). In contrast, helminth infections depend more directly on encounters with infective stages in the environment and patterns of fecal contamination (Nunn et al., 2011). Parasites with environmentally resistant eggs, such as *Trichuris*, may therefore persist and accumulate even in disturbed habitats where microbial exposure diversity declines. Disturbed environments may thus simultaneously reduce opportunities for microbial acquisition while maintaining or even increasing exposure to environmentally persistent helminths.

Understanding the interplay among social organization, habitat characteristics, and parasitic infection in wild primates deepens our ecological insights. It informs broader questions in evolutionary biology and conservation. The findings of this study underscore the complex, context-dependent nature of host–parasite dynamics. They highlight how intrinsic factors such as group size and behavior can mediate exposure risks, even in the face of external environmental change. Given the escalating habitat disturbance, a nuanced understanding is crucial for predicting disease risks in wildlife populations and for designing conservation strategies that account for social and ecological variability. Integrative approaches that consider behavioral ecology alongside environmental gradients may also be essential for safeguarding the health and persistence of socially complex yet increasingly vulnerable species such as the proboscis monkey.

Finally, the helminths detected in this study are of potential zoonotic relevance, especially in ecotourism landscapes such as the Menanggul River, where frequent contact occurs among humans, livestock, and wild primates. The genus *Trichuris* may include species closely related to *T. trichiura*, a well-known human pathogen that can infect non-human primates (Rivero et al., 2020). Likewise, *S. fuelleborni* and *O. aculeatum* have been reported in both human and non-human hosts in Southeast Asia (Yalcindag et al., 2021; Chan et al., 2023). However, because parasite identification in the present study was based solely on egg morphology, neither host specificity nor actual zoonotic transmission risk can be confirmed at this stage. Molecular characterisation will therefore be required to determine whether the detected parasites belong to human-infective lineages. Nevertheless, the presence of these helminths in areas accessible to humans highlights the need for further investigation of their transmission dynamics and potential public health relevance under increasing anthropogenic pressure (Gillespie and Chapman, 2006).

## Generative AI and AI-assisted technologies in the writing process

During the preparation of this manuscript, the authors used ChatGPT-5.2 to refine the clarity and logical flow of the text. The authors carefully reviewed, corrected, and approved all content generated, and take full responsibility for the final published version.

## CRediT authorship contribution statement

**Muhammad Nur Fitri-Suhaimi:** Writing – original draft, Methodology, Conceptualization. **Liesbeth Frias:** Writing – review & editing, Visualization, Methodology, Investigation, Formal analysis, Conceptualization. **Elke Zimmermann:** Methodology, Conceptualization. **Primus Lambut:** Writing – review & editing, Resources. **Joseph Tangah:** Writing – review & editing, Resources. **Henry Bernard:** Writing – review & editing, Resources. **Vijay Subbiah Kumar:** Writing – review & editing, Resources. **Ikki Matsuda:** Writing – original draft, Visualization, Validation, Supervision, Resources, Project administration, Methodology, Investigation, Funding acquisition, Data curation, Conceptualization.

## Ethics approval

The animal study was reviewed and approved by the Sabah Biodiversity Centre and the Sabah Wildlife Department [JKM/MBS.1000-2/2 JLD.3 (120)].

## Funding

This study was partially funded by the 10.13039/501100001691Japan Society for the Promotion of Science KAKENHI (nos. 24H00774 to I.M.; no. 23K27254 to G.H.) and Core-to-Core Program, Asia-Africa Science Platforms (JPJSCCB20250006 to IM).

## Declaration of competing interest

The authors declare no competing interests.

## Data Availability

All data generated or analyzed during this study are included in this published article.
